# The impact of an eight-week online physical activity intervention on sleep quality in early pregnancy: A pilot study using objective measurement

**DOI:** 10.18332/ejm/212552

**Published:** 2025-11-30

**Authors:** Summer S. Cannon, Melanie Hayman, Michele Lastella

**Affiliations:** 1 School of Health, Medical, and Applied Sciences, CQUniversity, Brisbane, Australia; 2 Appleton Institute, CQUniversity, Adelaide, Australia

**Keywords:** sleep, pregnancy, physical activity, wearable device

## Abstract

**INTRODUCTION:**

Sleep disturbances are common in pregnancy and linked to adverse maternal and fetal outcomes. Physical activity is a promising non-pharmacological strategy to improve sleep; however, few studies have objectively examined this relationship during early pregnancy. This pilot study examined whether participation in an eight-week, online, participant-centered physical activity program (Healthy Mamas Program) was associated with changes in objectively measured sleep among pregnant Australian women in their first trimester.

**METHODS:**

A prospective intervention study was conducted between March 2021 and November 2022. Eleven women who were not regularly active at baseline (<75 minutes/week) wore Fitbit Charge 2 devices to track daily physical activity and sleep. Participants completed a seven-day baseline assessment, followed by the eight-week intervention and a seven-day post-program assessment. Sleep and physical activity variables were transformed as appropriate. Descriptive statistics, Pearson correlations, and linear mixed-effects models were used to assess associations and changes over time.

**RESULTS:**

Participants engaged in 244.50 minutes of total physical activity per day, primarily of light intensity. No significant change in physical activity was observed over time (p=0.17). Participants averaged 5.9 hours of sleep per night, with 23.18 nightly awakenings and a mean sleep efficiency of 78.91%. There were no significant associations between physical activity and sleep duration (p=0.130) or efficiency.

**CONCLUSIONS:**

Findings suggest that, while feasible, the intervention did not significantly improve physical activity or sleep. Both behaviors remained below recommended levels. These results highlight the need for trimester-sensitive, accessible interventions to promote physical activity and improve sleep quality during pregnancy.

## INTRODUCTION

Pregnancy is a dynamic period of physiological and psychological transformation, marked by substantial biological, emotional, and behavioral changes^1^. Among the many challenges women encounter during this time are notable alterations in sleep patterns^2,3^. As gestation progresses, sleep disturbances become increasingly common, with many women reporting difficulty initiating and maintaining sleep, frequent nocturnal awakenings, and decreased sleep efficiency^2,3^. These disruptions are influenced by multiple physiological and psychosocial factors, including hormonal fluctuations (e.g. increased progesterone and estrogen), physical discomfort, urinary frequency, fetal movement, and anxiety about impending parenthood^1^.

Adequate sleep during pregnancy is essential – not only for maternal physical and psychological health, but also for optimal fetal development and pregnancy outcomes^2^. Despite this, up to 76% of pregnant women report poor sleep quality at some point during gestation, with evidence suggesting a progressive decline in sleep quality as pregnancy advances^3-5^. Poor sleep during pregnancy has been consistently linked to a range of adverse physiological and psychological outcomes. Physiological outcomes, include gestational hypertension, preeclampsia, gestational diabetes, prolonged labor, and an increased likelihood of cesarean delivery^2,3,6^. Psychologically, inadequate sleep is associated with a heightened risk of antenatal and postpartum depression, increased stress and anxiety levels, and impaired cognitive functioning^7-9^. For the fetus, maternal sleep disturbances have been linked to elevated risks of preterm birth, low birth weight, and potential neurodevelopmental delays^2^.

Given the prevalence and potential consequences of poor sleep during pregnancy, there is an urgent need for accessible, non-pharmacological strategies to improve maternal sleep. Pharmacological treatments are generally avoided in pregnancy due to potential fetal risks, making behavioral and lifestyle interventions the preferred approach^10^. Physical activity (PA) has emerged as a promising non-pharmacological strategy for improving sleep, in addition to its well-established benefits for pregnancy-related health outcomes, such as reduced risk of gestational diabetes and hypertensive disorders, improved psychological well-being, and enhanced cardiorespiratory fitness^11,12^. Systematic reviews suggest that PA can positively influence sleep quality, sleep duration, and sleep efficiency^13,14^. However, relatively few studies have objectively explored these associations in pregnant populations and even less among Australian mothers.

Despite these potential benefits, most pregnant women do not meet the recommended 150–300 minutes of moderate-to-vigorous PA per week^15,16^. Participation in PA often declines across pregnancy, particularly in the first trimester, when fatigue, nausea, and concerns about fetal safety are most prominent^17^. Additional barriers such as time constraints, lack of guidance, and physical discomfort further limit engagement in structured activity^17^.

Although PA has been increasingly recognized as a potential intervention for sleep disturbances, significant gaps remain in understanding its role in pregnancy. Much of the existing literature relies on self-reported data, which are susceptible to recall and social desirability biases, and lacks standardization in measurement tools and definitions of sleep quality^13,18^. Recent advances in wearable technology offer the ability to collect continuous, objective data on both sleep and PA in naturalistic settings^19,20^, yet such tools remain underutilized in pregnancy research.

Importantly, while PA and sleep have each been studied extensively as independent behaviors in pregnancy, their interrelationship remains underexplored. Evidence from the general population demonstrates that PA can improve sleep parameters, but it is unclear whether these effects extend to pregnant women, who experience unique physiological and behavioral constraints^18,21^. Understanding this relationship is critical to informing antenatal care practices and the development of effective interventions tailored to the needs of pregnant women.

Accordingly, the present study aimed to examine the relationship between PA and sleep quality among pregnant Australian women, using objective data collected via wearable devices. By leveraging continuous monitoring of both behaviors, this study sought to provide a more accurate and comprehensive understanding of how PA may influence sleep in pregnancy and to inform future intervention development.

## METHODS

### Study design

This study used a prospective, single-arm intervention design to objectively examine the relationship between PA and sleep during pregnancy. The study received ethics approval from the Central Queensland University Human Research Ethics Committee (approval number: 0000022021). The protocol was registered with the Australian New Zealand Clinical Trials Registry (ACTRN: 12620001074987). Data collection occurred between March 2021 and November 2022.

### Participants

Eligible participants were pregnant women aged ≥18 years, in their first trimester (up to 14 weeks gestation), who were not currently engaging in regular PA^13^. Participants were classified as inactive if they engaged in <75 min of moderate-intensity physical activity per week, consistent with previous research operationalizing inactivity as less than half of the internationally recommended 150 min per week for adults, including pregnant women^11,22,33^.

Participants were required to have sufficient English proficiency, access to a smartphone and internet, and no absolute or relative contraindications to PA based on current antenatal guidelines^14^. To determine possible contraindications, potential participants were asked to complete an online version of the Physical Activity Readiness Medical Examination for Pregnancy (PARmed-X for pregnancy)^24^. If participants answered ‘yes’ to any of the screening questions, they were asked to seek medical approval to participate in the study. Exclusion criteria included any absolute contraindications to PA (e.g. ruptured membranes, premature labor, placenta previa, or other uncontrolled medical conditions), or relative contraindications (e.g. excessive fatigue, vaginal bleeding, persistent headaches, or other significant medical issues)^14^. Recruitment involved the dissemination of study information using free and paid promotion of the study via social media. Participants provided informed consent prior to enrolment and could withdraw at any time.

### Measures

PA and sleep were objectively measured using Fitbit Charge 2 devices. The device captures continuous data using a 3-axis accelerometer and optical heart rate sensor. Fitbit devices have demonstrated acceptable validity for tracking activity intensity, sleep duration, and sleep efficiency when compared to gold-standard tools such as polysomnography and research-grade accelerometers^19,20^.

Sleep variables included sleep duration (min/night), sleep efficiency (%), number of awakenings (WASO), total time in bed, and wake after sleep onset. PA variables included total minutes active per day, and time spent in light-, moderate-, and vigorous-intensity activity based on Fitbit-defined MET thresholds (light: 1.5–2.7 METs; moderate: 2.8–4.3 METs; vigorous: >4.4 METs)^23,24^. Fitbit output was aggregated and analyzed at the daily and weekly levels.

### Intervention

Prior to beginning the intervention, participants completed a seven-day baseline assessment. During this time, they wore a Fitbit Charge 2 device continuously on their non-dominant wrist and completed three brief questionnaires on sleep quality, PA behavior, and well-being. No guidance or feedback was given during this baseline period. A mid-intervention follow-up occurred at Week 4, and a final post-intervention assessment took place during Week 8. These follow-ups included the same questionnaires and continued objective monitoring using Fitbit. Throughout the study, Fitbit data were synchronized to a secure server, de-identified, and stored in compliance with data protection protocols. Technical support was available for participants throughout the study. Resources for wellbeing support (e.g. Lifeline, Beyond Blue, Pregnancy, Birth, and Baby) were provided upon enrolment.

The Healthy Mamas Program was an eight-week, web-based intervention grounded in Social Cognitive Theory and aligned with Australian guidelines for physical activity during pregnancy^22,25^. Participants were provided with weekly instructions to progressively accumulate 50 min of MVPA in week 1, progressing to 75 min in week 2, 100 min in week 3, and 120 min in week 4. From week 5 onward, participants were prescribed 150 min of MVPA per week, in line with Australian guidelines^22^. This target was maintained through weeks 6–8. Recommended activities included walking, bodyweight exercises, and prenatal fitness videos available via the program website. The intervention incorporated key behavior change strategies including self-monitoring (via Fitbit and the Fitbit app), goal setting, personalized planning, and access to educational materials^25^.

The intervention was intentionally flexible to support individualized pacing based on symptoms and daily capacity. Participants had unlimited access to the Healthy Mamas website, which provided guided PA video sessions and PA information (guidelines and benefits). While behavioral strategies were embedded in the design, participant engagement metrics (e.g. website usage, video views) were not captured.

### Statistical analysis

All statistical analyses were conducted using IBM SPSS Statistics (version 30). Normality was assessed visually using histograms and formally using Shapiro–Wilk tests. Variables violating normality assumptions were transformed: square root transformations were applied to sleep duration, lightly active time, and number of awakenings; log transformations with a constant were applied to moderately and vigorously active minutes; and sleep efficiency was reflected (100-x) and square root transformed^19^.

Descriptive statistics summarized demographic and outcome variables. Pearson correlation coefficients were used to explore associations between PA and sleep variables. Linear mixed-effects models assessed changes in PA over time (fixed effect: week; random intercept: participant) and examined whether daily PA predicted nightly sleep outcomes. Significance was set at p<0.05.

## RESULTS

**Figure 1:**
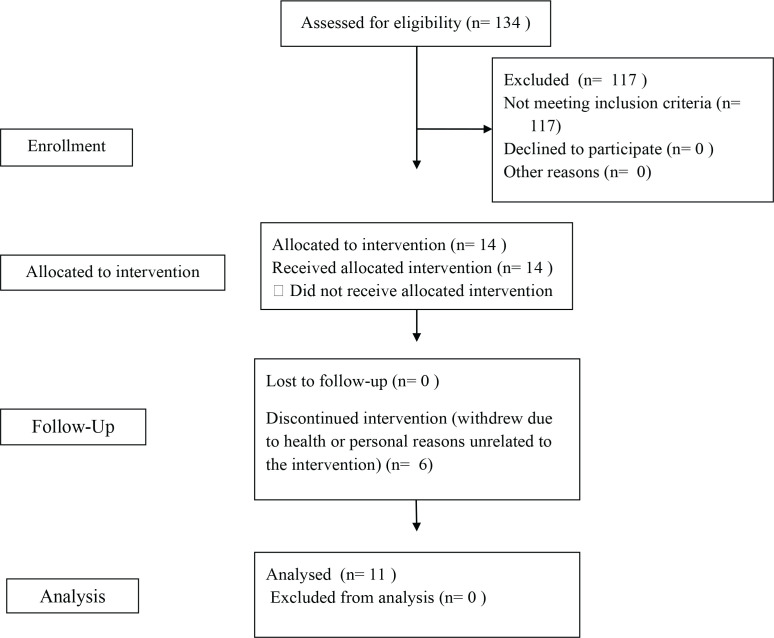
CONSORT 2010 flow diagram illustrating recruitment, enrolment, follow-up and analysis of participants in the Healthy Mamas Program (pilot online physical activity intervention), Australia, 2021–2022 (N=11)

A total of 134 individuals were screened for eligibility during the recruitment period. Of these, 114 were excluded due to not meeting the inclusion criteria. Seventeen participants met the inclusion criteria and enrolled in the study. However, six participants withdrew during the intervention period due to health issues or personal reasons (unrelated to their participation in the intervention itself), resulting in a final sample of 11 participants who completed the intervention. Participant flow through the study is presented in Figure 1. All participants were in their first trimester of pregnancy (gestational weeks 6–14) at the time of enrolment. The mean age of participants was 31.4 years (SD=5.82).

Results indicated no significant linear change in physical activity levels across the study period [F(1, 527.95)=1.88, p=0.171]. This suggests that participants’ total physical activity did not significantly increase or decrease over time. Physical activity data (Table 1) showed that the majority of movement was classified as light intensity (mean=233.26 min/day, SD=106.34), while moderate and vigorous activity levels were low (mean=7.46 min/day, SD=12.88; mean=3.77 min/day, SD=7.46; respectively).

**Table 1:** Mean daily minutes of light, moderate, vigorous, and total physical activity (PA), with estimated weekly minutes of moderate-to-vigorous physical activity (MVPA) across Weeks 1–8, pilot intervention study, Australia, 2021–2022 (N=11)

*Week*	*Light PA/day Mean (SD)*	*Moderate PA/day Mean (SD)*	*Vigorous PA/day Mean (SD)*	*Total PA/day Mean (SD)*	*Total MVPA (min/week)*
1	241.14 (114.82)	6.61 (12.62)	3.54 (7.14)	251.29 (114.62)	71.05
2	241.94 (112.37)	8.06 (14.44)	3.66 (7.11)	253.66 (117.67)	82.04
3	237.95 (100.88)	7.91 (11.66)	3.28 (5.83)	249.14 (103.13)	78.33
4	222.06 (122.26)	6.45 (10.84)	3.96 (7.81)	232.47 (123.23)	72.87
5	224.90 (88.59)	10.10 (18.38)	4.31 (6.57)	239.31 (89.25)	100.87
6	231.06 (78.09)	7.81 (15.46)	2.94 (4.90)	241.81 (81.51)	75.27
7	230.89 (95.67)	10.22 (13.55)	4.27 (7.46)	245.38 (95.39)	101.43
8	252.97 (98.91)	8.41 (11.14)	3.87 (7.93)	265.25 (104.82)	85.96

On average, participants obtained 354.32 min (approximately 5.9 h) (SD=142.57) of sleep per night, with a mean sleep efficiency of 78.91% (SD=17.73). The number of awakenings per night was high (mean=23.18, SD=12.90). No significant associations were found between total physical activity and minutes asleep [r(441)= -0.044; 95% CI: -0.137–0.050], or sleep efficiency [r(442)=0.019; 95% CI: -0.074–0.112]. These results indicate that, in this sample, daily physical activity was not strongly associated with either the duration or quality of sleep. Further, results indicated no significant association between total physical activity and sleep duration [F(1, 434.00)=2.30, p=0.130].

## DISCUSSION

This study investigated the effect of an eight-week online PA intervention (the Healthy Mamas Program) on sleep quality among pregnant women in their first trimester. Using objective, device-based measurement via wrist-worn Fitbit devices and a repeated-measures design, the study aimed to assess whether increased PA was associated with improvements in sleep variables. Contrary to expectations, no significant changes in PA were observed over time, and no associations emerged between PA levels and sleep parameters. Unlike much of the existing literature, which has relied on self-report measures, this study contributes novel evidence by integrating continuous, consumer-grade device monitoring within an intervention framework, offering insights into both feasibility and limitations of wearable technology in early pregnancy.

These findings may reflect both the modest intensity of the PA recorded and the physiological constraints of early pregnancy. Although participants accumulated some moderate-to-vigorous PA (MVPA) each week, average MVPA – as objectively measured via Fitbit – ranged 71–101 min/week, falling consistently short of the Australian guideline of ≥150 min/week during pregnancy^12^. The highest engagement (Week 7, mean=101.4 min/week) represented only 68% of the recommended target. While participants averaged 244.5 min of total daily PA, most of this was light-intensity movement (mean=233.3 min/day), with minimal time spent in moderate (mean=7.5 min/day) or vigorous activity (mean=3.8 min/day). These patterns were derived from continuous wrist-based accelerometry data and suggest that although participants were physically active, their movement was primarily incidental or of low intensity. When compared to existing literature, these findings are broadly consistent with reported PA levels among pregnant women^26^. Studies using objective methods have found that only a minority of pregnant women meet PA guidelines, with one Australian study reporting that just 32% of participants met MVPA targets in early pregnancy^15^. Internationally, similar trends are observed. For example, Evenson and Wen^27^ found that <25% of US pregnant women achieved recommended activity levels. This suggests that the low activity observed in the current cohort is typical, rather than abnormal, and may reflect common barriers in early pregnancy such as fatigue, nausea, and lack of tailored support^17,28^. However, the fact that participants in this study had consented to an PA program and were unable to reach guideline levels highlights a persistent gap between intention and behavior, underscoring the need for more targeted and supportive interventions during this period. This gap emphasizes the importance of designing interventions that go beyond providing information or resources, by incorporating trimester-sensitive tailoring and flexible digital strategies (e.g. adaptive exercise prescriptions, on-demand support) to help women overcome fluctuating energy levels and symptoms. Integrating flexible digital support, such as app-based feedback, symptom-adaptive modules, and opportunities for peer connection, may further strengthen engagement and adherence during this period^29^.

This is particularly relevant given that clinical benefits such as improved sleep are typically linked to moderate-intensity PA, rather than light PA alone^11,13,14^. The lack of sufficient intensity or variability in PA, as objectively measured, may therefore explain the absence of significant associations with sleep outcomes. Prior research supports this threshold effect, suggesting that light activity may be insufficient to elicit meaningful physiological or psychological benefits during pregnancy^11,30,31^.

In terms of sleep outcomes, participants averaged 354.3 min (approximately 5.9 h) of sleep per night and demonstrated a mean sleep efficiency of 78.9%, as measured by Fitbit’s proprietary sleep tracking algorithm^19,32^. Both values fall below thresholds generally recommended for maternal health: 7–9 h of sleep and sleep efficiency >85%^33,34^. Participants also experienced frequent nocturnal awakenings, which were objectively recorded by the wrist-worn devices. These findings are consistent with literature documenting poor sleep even in early pregnancy, likely reflecting common first-trimester complaints such as nausea, fatigue, hormonal shifts, increased urinary frequency, and anxiety^1,3,4,35^. For example, Sedov et al.^3^ conducted a meta-analysis that identified significant declines in sleep quality across pregnancy, including reductions in sleep duration and efficiency and increases in nocturnal awakenings, particularly in the first trimester. Their findings reinforce the interpretation that disrupted sleep is a normative experience in early pregnancy, influenced by both physiological changes and psychosocial stressors. Similarly, Mindell et al.^4^ observed that sleep duration tends to decline as pregnancy progresses, with notable increases in night-time awakenings even during the first trimester. Lu et al.^6^ further demonstrated that sleep disturbances in pregnancy are strongly associated with adverse maternal outcomes, underscoring the clinical significance of even modest reductions in sleep duration and efficiency. These complementary findings provide a broader context for understanding the persistent and multifactorial nature of poor sleep during early gestation.

The timing of the intervention is a critical contextual factor. The present intervention was introduced during the first trimester (6–14 weeks gestation) – a phase often characterized by fatigue, nausea, emotional lability, and reduced motivation^1,28^. Even with digital resources and personalized programming, these symptoms may have made it difficult for participants to engage in consistent, structured activity. This aligns with prior evidence showing that PA levels are lowest in the first trimester and may only stabilize or increase later in gestation^27^. Moreover, given the lack of increased PA during the intervention, it is not surprising that no improvements in sleep were observed. This finding aligns with prior literature indicating that sleep typically worsens as pregnancy progresses, and improvements are unlikely to occur without sufficient behavioral change^2,5,8^. Future research could directly compare the effectiveness of programs initiated in early versus later trimesters to determine whether timing influences feasibility, adherence, and outcomes.

### Limitations

Despite its strengths – including objective, continuous measurement of both PA and sleep via wearable devices and a repeated-measures design – this study has several limitations. The small sample size (n=11) substantially reduced statistical power, which may have limited the ability to detect statistically significant effects even if true effects existed. This limitation underscores the importance of replicating these findings in larger, more adequately powered trials to determine whether similar trends persist at scale. Prior research in behavioral intervention studies has emphasized the influence of statistical power on detecting small-to-moderate effects, particularly in health behavior change contexts where effect sizes are often modest^36^. Without adequate sample sizes, studies risk failing to identify real intervention effects, contributing to Type II errors and underestimation of potentially meaningful changes. Furthermore, engagement data (e.g. website usage, session completion) were not collected, restricting insight into adherence and dose-response effects. Additionally, while Fitbit devices offer practical, non-invasive monitoring, they remain consumer-grade tools with proprietary algorithms that may limit precision compared to research-grade actigraphy or polysomnography.

## CONCLUSIONS

Although this eight-week online PA program was well-structured and well-tolerated, it did not yield significant changes in objectively measured PA or sleep quality during early pregnancy. Both PA and sleep levels fell below recommended guidelines, with participants engaging in limited MVPA and experiencing suboptimal sleep duration and efficiency. These findings highlight the prevalence of low PA and poor sleep during pregnancy and the need for targeted, trimester-sensitive strategies to support maternal health. As a pilot study, the results should be interpreted as preliminary but provide valuable insights into the feasibility of digital PA interventions during early pregnancy. Future interventions should be adequately powered to detect change, tailored to the unique physiological and psychosocial challenges of each trimester, and designed to strengthen engagement through flexible digital support. Practical implications include trimester-sensitive tailoring of PA recommendations, flexible digital delivery, and exploration of strategies to enhance MVPA uptake in pregnant populations.

## Data Availability

The data supporting this research are available from the authors on reasonable request.
